# A cross-sectional investigation of parenting style and friendship as mediators of the relation between social class and mental health in a university community

**DOI:** 10.1186/s12939-015-0227-2

**Published:** 2015-10-05

**Authors:** Mark Rubin, Benjamin M. Kelly

**Affiliations:** The University of Newcastle, Callaghan, Australia

**Keywords:** Social class, Socioeconomic status, Parenting style, Friendship, Social integration, Mental health

## Abstract

**Introduction:**

This study tested a novel explanation for the positive relation between social class and mental health among university students. Students with a higher social class were expected to have experienced more authoritative and less authoritarian parenting styles; these parenting styles were expected to lead to greater friendship and social integration at university; and greater friendship and integration were expected to lead to better mental health.

**Method:**

To test this model, the researchers asked 397 Australian undergraduate students to complete an online survey. The research used a cross-sectional correlational design, and the data was analysed using bootstrapped multiple serial mediation tests.

**Results:**

Consistent with predictions, parenting style, general friendship and support, and social integration at university mediated the relation between social class and mental health.

**Conclusions:**

The present results suggest that working-class parenting styles may inhibit the development of socially-supportive friendships that protect against mental health problems. The potential effectiveness of interventions based on (a) social integration and (b) parenting style is discussed. Future research in this area should employ a longitudinal research design in order to arrive at clearer causal conclusions about the relations between social class, parenting styles, friendship, social integration, and mental health.

## Background

It is now well-established that social class and socioeconomic status (SES) are positively related to mental health [1–7]. For example, a meta-analysis of 51 studies found that people with a higher SES are less likely to be depressed than people with a lower SES [5]. However, researchers remain unclear about the specific processes that underlie the relation between social class and depression.

It is important to investigate social class differences in depression, mental health, and health in general because evidence-based interventions that reduce social class inequalities in mental and physical health can reap major economic benefits. For example, a recent report found that reducing the social class health inequality in Australia would result in savings of (a) $8 billion in extra annual earnings, (b) $3–4 billion per year in government pensions and allowances, (d) $2.3 billion per year in reduced health patient numbers, (e) $273 million per year in healthcare benefits, and (f) $185 million per year in the reduced use of prescribe medicines [8]. Hence, the potential cost savings from even modest reductions in social class health inequality are sizeable.

In the present research, we investigated a novel explanation of the relation between social class and mental health. We predicted that friendship, social integration, and parenting style mediate (account for) the relation between social class and mental health that occurs in university communities [9]. Below, we discuss the theoretical rationale and indirect evidence for this multiple serial mediator model. We begin by considering friendship and social integration as potential mediators of the relation between social class and mental health.

### Friendship and social integration

Friendship and social integration have beneficial effects on mental health and well-being [1, 10, 11]. From a theoretical perspective, this positive relation is most likely to occur because friendship and social integration facilitate social comparison, self-esteem, a sense of belonging, and perceived social support [12]. They also protect against mental illness by buffering the effects of stressful events [13, 14]. Notably, a reverse causal relationship is also possible: Poorer mental health may result in fewer friends and less social integration due to the social stigma attached to mental illness [15].

Previous research has also found that middle-class people tend to have larger social networks than working-class people [2, 16–19]. Furthermore, middle-class students are more socially integrated at university than working-class students [20].

Given that friendship and social integration positively predict mental health, and that working-class people have smaller social networks and are less integrated at university than middle-class people, it is possible that social class differences in mental health result from social class differences in friendship and social integration [21–23]. However, there is currently no conclusive evidence to support this mediation effect. Most previous research that has measured SES, social network size and support, and mental health has not investigated the mediation effect that we have proposed [1, 17]. The exception is a study that found that lower SES people tended to have less social support and were more likely to be depressed, but that SES differences in social support did not account for SES differences in depression [2]. However, the researchers of this study conceded that their “assessments and classifications of support were rather brief and crude” (p. 62). Hence, this null mediation effect may be attributed to insensitive measures of friendship and support rather than a genuine null mediation effect. The present research used more comprehensive measures of friendship, social support, and social integration in order to provide a more sensitive test of their potential mediating role.

### Parenting style

The present research also provided a more nuanced investigation by considering *why* people with higher social class might have greater friendships and experience greater social integration. We proposed that parenting style may help to explain this relation.

Parenting style refers to the ways in which parents interact with their children [24]. Two key styles have been contrasted in the literature: The *authoritative* style is characterized by high degrees of warmth and responsiveness by parents towards their children. In contrast, the *authoritarian* style is characterized by less warmth and a more restrictive, disciplinary, and controlling approach. The parenting style that people experienced as children may mediate the relation between their social class, friendship and social integration, and mental health because it is related to each of these variables.

First, parenting style is related to social class: Working-class parents tend to have a less authoritative and more authoritarian parenting style than middle-class parents [25–33]. These social class differences may exist because working-class parents have less autonomy in their jobs, fewer financial resources, and more disorder in their neighborhoods. Consequently, they are less skilled and less able to adopt the more creative and resource-intensive authoritative parenting style [32, 34]. In addition, working-class parents value conformity and middle-class parents value self-direction, and these social class differences in values prescribe authoritarian and authoritative parenting styles respectively [35, 36]. Finally, working-class parents tend to live in neighborhoods that expose them and their children to greater threats and risks that activate parental protection mechanisms which produce less authoritative and more authoritarian parenting styles [37].

Second, parenting style is related to students’ adjustment at university. In particular, authoritative parenting predicts better academic, social, and personal-emotional adjustment at university as well as a greater sense of attachment to the institution [38]. This greater adjustment may be because authoritative parenting promotes greater self-management, promotion-focused self-regulation, social competence, optimism, theory of mind, and successful interpersonal relations [38–43]. All of these psychological qualities are beneficial for developing friendships and integrating in social groups.

Finally, parenting style predicts children’s mental health and psychosocial well-being [43–45]. In particular, authoritative parenting positively predicts self-esteem and optimism and negatively predicts anxiety and depression [38–40, 46–50]. In contrast, authoritarian parenting negatively predicts self-esteem and emotional well-being and positively predicts anxiety, depression, sense of inadequacy, symptomatic problems, risk to self and others, and suicidal ideation [39, 48, 50–52].

Despite the established relations between parenting style and (a) social class, (b) students’ adjustment at university, and (c) mental health and well-being, no previous research has considered parenting style as a potential mediator of social class differences in mental health. The current research addressed this important and innovative research question.

### Overview of the present research

**Fig. 1:**
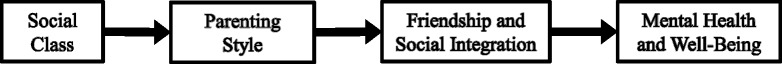
The effect of social class on mental health via parenting style and friendship and social integration

Given the theoretical and empirical relations between social class, parenting style, friendship and social integration, and mental health and well-being, it is plausible that parenting style and friendship and social integration mediate the relations between social class and mental health and well-being. This multiple serial mediation model is presented in Fig. 1.

We investigated this previously-untested model in the context of a university community. Here, we predicted that that students from higher social class backgrounds have experienced a more authoritative and less authoritarian parenting style, and that these parenting styles encourage the development of a range of socially-beneficial psychological resources (e.g., self-management, promotion-focused self-regulation, social competence, theory of mind) that enable students to develop greater friendships and social integration at university. In turn, better friendships and social integration were expected to lead to better mental health and well-being due to their stress-buffering effects and beneficial effects on self-esteem, sense of belonging, and perceived social support.

It is important to investigate the potential mediating roles of parenting style and friendship and integration in explaining the relations between social class and mental health because this type of research can inform the development of social interventions that improve the mental health of people from working-class backgrounds. In particular, our proposed model suggests two potential interventions: (a) proximal interventions that improve the social integration of working-class people and (b) distal interventions that encourage working-class parents to adopt more authoritative and less authoritarian parenting styles. We discuss these potential interventions in greater detail in the Implications section.

## Method

### Participants and design

The research used a cross-sectional correlational design and quantitative self-report measures that were presented in an online survey. Participants were undergraduate psychology students at a large public Australian university. A large (*N* = 6044) cross-sectional survey that was conducted in 2010 confirmed that students at this university who had low incomes (indicated by their possession of a government healthcare card) were more likely to experience depression and anxiety [53]. Hence, the relation between social class and mental health was clearly evident in this population.

The university had 27.32 % low SES students based on students’ residence in low SES locations. This figure was higher than average at Australian universities (15.95 %) but representative of the percentage of low SES people in the Australian population (~25 %).

Ethical approval for the research was obtained from the university’s Human Research Ethics Committee (H-2012–0382). The study was advertised in a list of other research studies via an online research participant pool system that was based within the psychology department. Participants were free to decide whether or not to complete the research. They were awarded 1.0 % course credit for taking part in the study.

We performed an a priori power analysis in order to establish our sample size. A recent meta-analysis found that the relation between social class and the prevalence of depression was represented by an overall odds ratio of 1.81 with a 95 % CI of 1.57–2.10 [5]. This odds ratio is equivalent to an effect size of *r* = −.16. We calculated that 406 participants are required in order to detect an effect size of this magnitude using a two-tailed bivariate correlation test with an alpha level of .05 and a power value of .90. In the present research, this sample size was rounded up to 410 participants in order to account for potential participant withdrawals and the exclusion of outliers in the data.

We collected data from 410 participants. Of these, 13 participants indicated that they did not want their data to be included in the data analyses. These 13 exclusions left a total of 397 participants.

There were 321 women (80.86 %) and 76 men (19.14 %). This underrepresentation of men is typical in undergraduate psychology programs. To address this gender imbalance, we included gender as a covariate in our analyses in order to control for potential gender effects.

Participants ranged in age from 17 to 51 years with a mean age of 21.94 (*SD* = 6.51). The majority of participants self-identified as Caucasian (89.0 %). The remainder self-identified as other (4.86 %), Aboriginal (2.81 %), Asian (2.81 %), or African (.51 %).

Based on the measure of social class identity (see below for details), 13.85 % of participants described themselves as “working-class,” 6.80 % as “lower middle-class,” 40.05 % as “middle-class,” 30.98 % as “upper middle-class,” .99 % as “upper-class,” and 6.30 % indicated that they did not know their social class.

### Measures

#### Social class

Following previous researchers, we used a selection of widely-used measures of social class [3, 54, 55]. These measures included assessments of parental education, occupation, income, social class identity, and house size. Participants indicated the highest education level of (a) their mother and (b) their father using the following categories: *no formal schooling, primary school (kindergarten to year 6), secondary or high school (years 7 –10), senior secondary school (years 11 & 12), technical and further education (TAFE), university - undergraduate degree (Bachelor degree), university - postgraduate degree (Masters or PhD)*. They also indicated how they thought most people would rate the occupation of (a) their mother and (b) their father in terms of its prestige and status on an 11-point scale anchored *extremely high status and prestige* and *extremely low status and prestige*. Participants indicated their family income during childhood using a 5-point scale anchored *well above average* and *well below average*. They also indicated the social class that they felt best described (a) themselves, (b) their mother, and (c) their father using a 5-point scale: *working class, lower middle-class, middle-class, upper middle-class, upper class* [56]. Finally, participants indicated the number of bedrooms in their parents’ house when they were 15 years old using an 11-point scale anchored *one *and *more than ten*.

#### Parenting style

We measured parenting style using items that were adapted from the Parenting Behavior Questionnaire - Head Start (PBQ-HS; [57]), which is a modified version of the Parenting Behavior Questionnaire [58]. Following previous researchers [41], we used 13 of the 16 items from the PBQ-HS active responsive subscale and all nine items from the active restrictive subscale. The active responsive subscale assesses parental warmth, responsiveness to children’s needs, and respect for children’s autonomy, which are the key aspects of authoritative parenting [57]. The active restrictive subscale assesses the demands that parents place on their children and the use of criticism and punitive discipline, which are the key aspects of authoritarian parenting.

Following previous researchers in the area, we adapted the items in the PBQ-HS in order to allow respondents to report on their parents’ behavior [38, 41]. Hence, an example item from the active responsive style subscale is “my parents told me that they were proud of me when I was trying to be good at something.”

#### Friendship and social integration

We measured friendship and social integration using a combination of scales that assessed friendship quality, loneliness, social support, sense of belonging, and community participation. The scales included the 8-item Friendship Goals scale [59], the 20-item Revised UCLA Loneliness Scale [60], the 6-item Friendship Scale [61], the 4-item Guidance subscale of the Social Provisions Scale [62], a 6-item version of the Sense of Belonging scale [63], and a version of the 5-item Community Participation subscale of the Perceived Community Support Questionnaire that was adapted to refer to the university community [64]. We also included an ad hoc 3-item measure of relationship closeness and satisfaction at university that included the following items: “I am satisfied with my social life at the university,” “I feel close to my friends at the university,” and “I am satisfied with the quality of the relationships that I have with my university friends.”

#### Mental health

We included the following measures of mental health in our survey: the 21-item Beck Depression Inventory-II [65], the 21-item Beck Anxiety Inventory [66], the 21-item Depression Anxiety Stress Scale [67], and the 25-item Depression Happiness Scale [68]. We also included a measure of well-being: the 5-item Satisfaction with Life Scale [69].

### Procedure

Participants completed an online survey from any computer that had internet access. The survey was titled “Parents, Personality and Feelings,” and participants read that it was investigating “the role parenting has in the development and expression of aspects of personality and feelings.”

Participants were asked to complete the survey in their own time, on their own, and in an environment that did not contain any distractions. Participants took a median time of 23.00 min to complete the survey.

The previously-described scales were presented in a random order for each participant with the exception of the social class measures, which were always presented near the end of the survey in order to avoid cuing participants to the relevance of social class prior to their completion of the outcome and mediator variables. Items within each scale were presented in a random order for each participant.

At the end of the survey, participants typed what they thought the research was trying to show and how it was trying to show it. Most participants reiterated the information with which they had already been provided; that the study was investigating the relation between parenting, personality, and feelings. Only a few participants mentioned “social class” or “socioeconomic status” (*n* = 12; 3.02 % of the sample), indicating that this aspect of the study was not particularly salient to our participants.

## Results

### Exploratory factor analyses

Conducting separate statistical tests on our nine measures of social class, seven measures of friendship and social integration, and five measures of mental health would greatly increase the risk of obtaining spurious results due to Type I (false positive) errors. In order to reduce this risk, we investigated the possibility of combining some of our measures into higher-order aggregate indices that would provide more reliable and sensitive assessments of our key constructs than their individual constituent measures.

#### Social class

We investigated whether it was appropriate to combine the nine measures of social class into a single global index [20, 54, 55]. The nine measures of social class (i.e., highest education of mother and father, occupational status of mother and father, family income during childhood, social class identity of mother, father, and self, number of bedrooms in parent’s house when 15 years old) were converted to *z* scores in order to produce comparable metrics. They were then included in a principal axis exploratory factor analysis.

To determine the number of factors to extract, we conducted a parallel analysis [70] using Monte Carlo simulation software [71]. We simulated factor analyses on 500 random data sets, each comprising 9 variables and 397 participants. The results showed that only the first two factors in the real data set had eigenvalues that were larger than the first two factors in the simulated data sets (real eigenvalues: 3.83 & 1.20; simulated eigenvalues: 1.24 & 1.16). Consequently, we specified the extraction of two factors using a promax rotation in order to allow the factors to be correlation with one another.

All of the social class items loaded at .40 or greater on the first factor apart from the number of bedrooms question (.37) and mother and father’s highest level of education (−.17 & .31 respectively). The only item to load more highly on the second factor than on the first was mother’s highest level of education (1.06). Based on these results, we proceeded to create an aggregate index of social class that included all of the social class items apart from number of bedrooms and mother and father’s highest level of education. These six social class items had a good mean correlation with one another (*r* = .47) and a good internal reliability (Cronbach α = .77).

#### Friendship and mental health

We also conducted a principal axis exploratory factor analysis on *z* score transformations of the friendship and mental health variables (i.e., Friendship Goals scale, Revised UCLA Loneliness Scale, Sense of Belonging scale, community participation at university, relationship satisfaction and closeness at university, Friendship Scale, Guidance scale, Beck Depression Inventory-II, Beck Anxiety Inventory, Depression Anxiety Stress Scale, Depression Happiness Scale, and Satisfaction with Life Scale). To identify the number of factors for extraction, we simulated factor analyses on 500 random data sets, each comprising 12 variables and 397 participants. Only the first three factors in the real data set had eigenvalues that were larger than the first three factors in the simulated data sets (real eigenvalues: 6.06, 1.42, & 1.12; simulated eigenvalues: 1.21, 1.16, & 1.10). Consequently, we specified the extraction of three factors.

All of the mental health scales loaded at .73 or greater on the first factor apart from satisfaction with life, which loaded at –.38. Hence, the first factor represented mental health.

The Revised UCLA Loneliness Scale, Guidance scale, Friendship Scale, and Friendship Goals scale all loaded at .48 or above on the second factor. We conceptualized this second factor as a measure of general friendship and social support. The Satisfaction with Life scale also loaded at .42 on this second factor. However, given that satisfaction with life is conceptually different from general friendship and support, and given that it also loaded substantially on the mental health factor (−.38), we excluded this variable from the aggregate indices that represented either construct.

Sense of belonging at university, community participation at university, and relationship satisfaction and closeness at university all loaded at .56 or higher on the third factor. Hence, this third factor represented social integration at university.

Based on the above analyses, we created three higher-order variables. The *mental health* index was the mean of the *z*-score transformed scores from the Beck Depression Inventory-II, Beck Anxiety Inventory, Depression Anxiety Stress Scale, and Depression Happiness Scale (interitem *r* = .73, Cronbach α = .91). To aid interpretation, we reverse-scored this index so that *higher* scores indicated *better* mental health rather than *worse* mental health.

The *general friendship and support* index was the mean of the *z*-score transformed scores from the Friendship Goals scale, Revised UCLA Loneliness Scale, Friendship Scale, and Guidance scale (interitem *r* = .49, Cronbach α = .79). Finally, the *social integration at university* index was the mean of the *z*-score transformed scores from the sense of belonging at university, community participation at university, and relationship satisfaction and closeness at university scales (interitem *r* = .50, Cronbach α = .75).

### Zero-order correlation analyses

Table 1 presents the zero-order correlation coefficients for the relations between the key variables.

**Table 1:** Zero - order correlation coefficients

Measure	1	2	3	4	5	6
1. Social class	–					
2. Authoritative parenting	.30**	–				
3. Authoritarian parenting	–.25**	-.43**	–			
4. General friendship and support	.20**	.43**	–.16**	–		
5. Social integration at university	.22**	.25**	–.09	.50**	–	
6. Mental health	.15**	.31**	–.14**	.60**	.44**	–
7. Satisfaction with Life	.24**	.42**	–.25*	.62**	.43**	.66**

*p < .05. **p < .01. *N* = 397 apart from for the correlations in Column 1, where *N* = 393 due to missing data in the social class variable

Consistent with previous research, social class a small but significant positive relations with mental health and well-being: Students with higher social class experienced better mental health and well-being. Also consistent with previous research, social class had medium-size relations with parenting style: The higher students’ social class, the more authoritative and less authoritarian they reported their parents to be. Finally, social class had small-to-medium size positive relations with (a) general friendships and support and (b) social integration at university: Students with higher social class reported greater friendship and support and greater social integration at university.

Table 1 also shows that parenting style was significantly related to mental health and well-being. Authoritative parenting had medium-size positive relations with mental health and satisfaction with life, and authoritarian parenting had small-to-medium size negative relations with these variables. In addition, parenting style was related to friendship and social integration: More authoritative and less authoritarian parenting were related to greater friendship and support and greater social integration at university, although these effects were weaker for authoritarian parenting than for authoritative parenting, and they fell below the conventional level of significance in the case of the relation between authoritarian parenting and social integration (*r = −*.09, *p* = .082).

Finally, general friendships and social integration showed medium-to-large size positive relations with mental health and well-being: Greater friendships and support and better social integration at university were associated with better mental health and greater satisfaction with life.

### Mediation analyses

We predicted that parenting style and friendship and social integration mediate the relations between social class and mental health. To test this multiple serial mediation model, we used PROCESS software [72]. PROCESS uses a path analytical framework to estimate direct and indirect effects in mediator models. Unlike structural equation modelling, PROCESS uses a bootstrapping approach to provide powerful estimates of direct and indirect effects, and it allows the more precise computation of serial indirect effects.

We used PROCESS Model 6, which tests the indirect effect of a predictor variable (social class) on an outcome variable (mental health or well-being) via a series of mediator variables (parenting style, general friendship and support, social integration at university) that are assumed to operate in a serial manner (i.e., each mediator transmits the effect to the next in the chain). In designing our models, we positioned general friendship and support before social integration at university in the mediation chain based on the reasonable assumption that students’ general friendship and support are more likely to influence their social integration at university rather than vice versa. Table 2 presents the results of our mediation tests.

**Table 2:** Results of multiple serial mediation tests

		Indirect effect		Total effect		Direct effect	
Parenting	Outcome	*b*	Bootstrapped	*b*	*p*	*b*	*p*
Style	variable	(*SE*)	95 % CIs	(*SE*)	(95 % CIs)	(*SE*)	(95 % CIs)
Authoritative	Mental	0.0133	0.0065, 0.0243	0.180	.002	0.002	.971
	health	(0.0043)		(0.059)	(0.065, 0.295)	(0.049)	(−0.099, 0.095)
Authoritarian	Mental	0.0032	0.0005, 0.0091	0.180	.002	0.003	.950
	health	(0.0021)		(0.059)	(0.065, 0.295)	(0.049)	(−0.094, 0.100)
Authoritative	Satisfaction	0.0153	0.0041, 0.0328	0.444	< .001	0.118	.127
	with life	(0.0070)		(0.093)	(0.261, 0.627)	(0.077)	(−0.034, 0.270)
Authoritarian	Satisfaction	0.0038	0.0004, 0.0122	0.444	< .001	0.128	.098
	with life	(0.0027)		(0.093)	(0.261, 0.627)	(0.077)	(−0.024, 0.280)

All tests are multiple serial mediation tests in which social class predicts either mental health or well-being via parenting style (authoritative or authoritarian), general friendship and support, and social integration at university, in that order. The first two columns indicate the two variables that change between tests: parenting style (authoritative or authoritarian) and the outcome variable (mental health or well-being). The indirect effect column presents the serial multiple mediation effects. The total effect column presents the effects of social class on the outcome variables without controlling for any of the mediator variables. The direct effect column presents the effect of social class on the outcome variables when controlling for the mediator variables. All beta values are unstandardized coefficients. 95 % CIs = the upper and lower 95 % confidence intervals, *SE* = standard errors. The reported tests do not include gender, age, or ethnicity as covariates

Our first mediation model included social class as the predictor variable, authoritative parenting style (i.e., the PBQ-HS active responsive subscale), general friendship and support, and social integration at university as the mediator variables, and mental health as the outcome variable. Consistent with the zero-order correlations, the total effect of social class on mental health was positive and significant. In addition, the direct effect of social class on mental health when controlling for the three mediators was nonsignificant. In order to estimate the reliability of the associated serial indirect effect (i.e., the total effect minus the direct effect), we used 5000 bootstrapping iterations to obtain bias-corrected and accelerated bootstrap 95 % confidence intervals. Confirming a significant full mediation effect, the serial indirect effect was significant (i.e., the two 95 % confidence intervals did not fall either side of zero).

Our second mediation test replaced authoritative parenting with authoritarian parenting style (i.e., the PBQ-HS active restrictive subscale) as a mediator. Again, the total effect of social class on mental health was significant, the direct effect controlling for the three mediators was nonsignificant, and the serial indirect effect was significant.

We also investigated similar mediation models using satisfaction with life as the outcome variable instead of mental health. When authoritative parenting was included as the first of the three mediators, the total effect of social class on satisfaction with life was significant, the direct effect controlling for the three mediators was nonsignificant, and the serial indirect effect was significant. Similarly, when authoritarian parenting was included as the first of the three mediators, the total effect was significant, the direct effect was nonsignificant, and the serial indirect effect was significant. Hence, parenting style and friendship and social integration mediated the relation between social class and well-being as well as between social class and mental health.

We tested all of our mediation models with and without outliers and with and without gender, age, and ethnicity included as covariates. Gender was included as a covariate because there was a gender imbalance in our sample (80.86 % women). Age was included because prior research has shown that age differences can account for social class differences in friendships in higher education [54]. Finally, ethnicity was included because prior research has shown that ethnicity often covaries with social class [73]. The inclusion and exclusion of outliers and covariates did not produce any substantial differences in results.

## Discussion

The present research makes three unique contributions to the literature in this area. First, it provides evidence that friendship and social integration mediate the relation between social class and mental health. This mediation effect is most likely to occur because friendship and social integration provide self-esteem, a sense of belonging, and social support, which are all beneficial for mental health, and working-class people tend to have fewer friends and be less socially integrated than middle-class people. Previous tests of the mediating roles of friendship and social integration have not been conclusive [2]. The present research highlights the importance of using more comprehensive measures of friendship, social support, and social integration in order to provide sensitive tests of their mediating role.

Second, the present research provides the first evidence that parenting style mediates the relation between social class and mental health. This mediation effect is most likely to occur because the parenting styles that people experienced during their childhood predict their subsequent degree of friendship and social integration, and working-class people tend to experience parenting styles that are less beneficial for the development of friendships and social integration.

Third, the present research provides the first evidence for a multiple serial mediator model that explains the relations between social class and mental health and well-being by considering parenting style and friendships and social integration as serial mediators. Specifically, the present research found that (a) undergraduate students’ social class predicted their parents’ authoritative and authoritarian parenting styles during their childhood and adolescence, (b) that these parenting styles predicted students’ general friendships and support, (c) that friendship and support predicted students’ social integration at university, and (d) that this social integration predicted students’ mental health and well-being. Hence, the higher students’ social class, the more authoritative and less authoritarian their parents’ parenting style, the greater their friendships and social integration at university, and the better their mental health and well-being. More simply, parenting style and friendship and social integration mediated the relation between social class and mental health and well-being. To our knowledge, this is the first evidence in support of this multiple serial mediation model.

Importantly, the present cross-sectional correlational research design does not allow us to draw clear causal conclusions regarding the relations that we observed. Nonetheless, the observed effects are consistent with our proposal that students with a higher social class experience a more authoritative and less authoritarian parenting style that results in greater friendship and social integration at university and, consequently, better mental health and well-being. Conversely, working-class parenting styles appear to inhibit the development of socially-supportive friendships that protect against mental health problems at university.

### Limitations and future directions

As noted above, a key limitation of the present research is that it used a cross-sectional correlational design. Future research should use a longitudinal research design in order to reach firmer conclusions regarding the causal direction of the effects that we observed. Having said this, even within the limits of a cross-sectional design, we can be more confident about the causal direction of some effects than of others. In particular, it is more plausible that social class influences parenting style than vice versa. Similarly, it is more reasonable to assume that parenting style influences friendships and social integration than vice versa. However, it is the case that people with mental health problems find it more difficult to make friends and integrate [15]. Hence, it is possible that the students in our sample who had mental health problems had more difficulty integrating at university and making friends in general because of these problems. A longitudinal research design would allow researchers to measure general friendship, social integration, and mental health at two time points and demonstrate that changes in friendship and social integration over time are responsible for subsequent changes in mental health. This type of research is required in order to confirm our assumption that greater friendships and social integration *cause* better mental health.

A second limitation of our research relates to our research population. Our research participants were Australian psychology undergraduate students who were enrolled at a specific public university, and women represented 80.86 % of our sample. It is encouraging that previously-demonstrated effects between social class, parenting style, friendship and social integration, and mental health and well-being all generalized to this specific sample. These replications add to a growing body of evidence that indicates that the relations between these variables are relatively robust and widespread. Nonetheless, future research should test the generality of our multiple serial mediation effect in alternative populations and communities.

A further limitation relates to our measure of parenting style. We used the common approach of asking respondents to report on their parents’ behavior [37, 40]. However, this approach may be susceptible to recall or reporting biases. A more direct and potentially more reliable and valid approach would be to ask parents to report on their own parenting styles. In addition, we did not include measures of *permissive parenting* in the present research. Permissive parenting has been characterized as a warm, accepting, and uncontrolling attitude towards the child [24]. However, there is some debate over the defining characteristics of permissive parenting [28, 57]. Furthermore, although a negative relation has been predicted between social class and permissive parenting [57], the evidence is mixed, with some research finding a negative relation [25, 28] and other research finding a positive relation [74]. Future research should explore the relations between social class, permissive parenting, and mental health.

Future research should also measure a greater number of variables that covary with social class, parenting style, friendship, social integration, and/or mental health and well-being. In the present research, we included gender, age, and ethnicity as covariates. Future research should also include variables such as grade point average, current level of financial hardship, neighborhood quality, adverse child events, and risk exposure. For example, researchers have found that multiple risk exposure helps to explain the positive relation between SES and mental and physical health (for a review, see [75]).

Finally, it is important to establish the extent to which our findings generalize outside of a university context. Working-class status is likely to be particular salient in university contexts, and there are likely to be fewer opportunities for associating with other working-class people at university than there are in other contexts. Future research should investigate the extent to which similar processes to the ones that we have observed operate in communities other than university communities.

### Implications

University students report disproportionately high levels of mental distress relative to the general population. For example, a study conducted at two large Australian universities found that 19 % of students had “very high” levels of mental distress, whereas only 3 % of the general population had equivalent levels of distress [76]. Similar results have been obtained at American universities [77]. Moreover, working-class students appear to be particularly susceptible to mental health problems at university [9]. Consequently, there is a pressing need to understand the processes that are responsible for mental distress among working-class university students. The present research addresses this need by demonstrating that the positive relation between social class and mental health is mediated by parenting style and general and university-specific friendship and social integration.

Although it is too early to draw firm causal conclusions about the relationships that we have identified, the present preliminary results suggest two potential interventions for reducing social class differences in mental health in university communities and, potentially other communities if our effects generalize to these communities. The first, more proximal intervention relates to social integration. If working-class students suffer worse mental health than middle-class students due to their poorer social integration at university, then a potential solution is to increase their social integration at university. Hence, potential interventions may include greater opportunities for on-campus accommodation, on-campus social activities, and on-campus paid employment for working-class students [54, 55].

The second, more distal intervention relates to parenting style. If working-class people suffer worse mental health than middle-class people due to their exposure to a less authoritative and more authoritarian parenting style, then a potential solution is to intervene to alter the working-class parenting style to make it more authoritative and less authoritarian. If working-class parents adopt more authoritative and less authoritarian parenting styles, then their children may grow up to enjoy greater friendship and social integration and, consequently, better mental health and well-being. However, it is important to appreciate that any such parenting style intervention needs to take into consideration the impact of an array of sociocultural factors. To illustrate, we briefly consider social context, historical context, and cultural context.

First, the relation between parenting style and mental health needs to be considered within specific social contexts. For example, it has been proposed that authoritarian parenting may actually benefit children who live in harsher social environments (e.g., rundown inner-city environments) because it teaches them to protect themselves against threats from others [41, 78].

Second, culturally-prescribed beliefs about parenting change over the years. Hence, although once popular, authoritarian parenting is receiving less and less endorsement in contemporary society [33, 35, 79]. It may be that the negative outcomes of authoritarian parenting are less to do with the intrinsic nature of this style and more to do with the minority-based discrimination that children experience because they are different from the majority group of authoritatively-parented peers.

Third, although the evidence is mixed, some studies show that the relation between parenting style and mental health varies across cultures. For example, a study of Egyptian adolescents concluded that “authoritarian parenting within an authoritarian culture is not as harmful as within a liberal culture” [80] (p. 103).

Hence, although the present results indicate that parenting style and friendship and social integration are key mediators of the relation between social class and mental health and well-being, these processes are liable to be heavily influenced by the specific sociocultural contexts in which they occur, and parenting style interventions that are intended to leverage these processes to improve mental health need to be carefully framed within these contexts.

## Conclusions

The present research provides the first empirical demonstration that parenting style, friendship, and social integration mediate the positive relations between social class and mental health and well-being in a university community. Specifically, the research shows that, compared to middle-class students, working-class students report that their parents had more authoritarian and less authoritative parenting styles. These social class differences in parenting styles predicted social class differences in general friendships and social integration at university that, in turn, predicted social class differences in mental health and well-being.

This new evidence is consistent with the view that working-class parenting styles inhibit the development of socially-supportive friendships that protect against mental health problems at university. However, the cross-sectional correlational research design that was used in the present research precludes clear causal inferences about the relationships that we observed. Additional longitudinal evidence is required in order to arrive at firmer conclusions in this regard. What is clear is that parenting style has a hitherto unacknowledged role to play in explaining the positive relation between social class and mental health. Furthermore, interventions that are intended to reduce social class inequity in mental health need to consider both parenting style and friendship and social integration in their design.
